# CircRFX3 contributes to glioma progression through the circRFX3-miR-1179/miR-1229-VASP axis

**DOI:** 10.1186/s12935-021-02293-0

**Published:** 2021-11-02

**Authors:** Hongli Li, Yiwei Zhang, Huiqin Song, Li Li

**Affiliations:** 1 Department of Neurology, Huaihe Hospital, Henan University, Kaifeng, Henan China; 2 School of Nursing and Health, North Section of Jinming Avenue, Henan University, Kaifeng, 475001 Henan China; 3 Department of Pathology, The First Affiliated Hospital of Henan University, Kaifeng, Henan China

**Keywords:** Glioma, circRFX3, miR-1179, miR-1229, VASP

## Abstract

**Background:**

Circular RNAs (circRNAs) are implicated in the carcinogenesis of human cancers. However, the functional roles of circRFX3 in glioma are not elucidated.

**Methods:**

Quantitative real-time polymerase chain reaction (qRT-PCR) assay was performed for the levels of circRFX3, RFX3, miR-1179, miR-1229 and vasodilator stimulated phosphoprotein (VASP). Actinomycin D assay and RNase R assay were employed to analyze the characteristics of circRFX3. Cell Counting Kit-8 (CCK-8) assay and colony formation assay were conducted for cell proliferation. Transwell assay was used for cell migration and invasion. Flow cytometry analysis was adopted for cell apoptosis. RNA pull-down assay, dual-luciferase reporter assay and RNA immunoprecipitation (RIP) assay were employed to analyze the interaction between miR-1179/miR-1229 and circRFX3 or VASP. Western blot assay was conducted for VASP protein level. Murine xenograft model assay was used to investigate the role of circRFX3 in vivo.

**Results:**

CircRFX3 level was increased in glioma tissues and cells. Knockdown of circRFX3 suppressed glioma cell proliferation, migration and invasion and promoted apoptosis in vitro and repressed tumorigenesis of glioma in vivo. MiR-1179 and miR-1229 were identified to be the targets of circRFX3. MiR-1179 or miR-1229 inhibition reversed the impacts of circRFX3 knockdown on glioma cell malignant behaviors. Additionally, VASP was demonstrated to be the target gene of miR-1179 and miR-1229, and VASP overexpression abolished the effect of circRFX3 knockdown on glioma cell progression.

**Conclusion:**

CircRFX3 served as a tumor promoter in glioma via modulating miR-1179/miR-1229-VASP axis, which might provide a novel target for glioma therapy.

## Introduction

Glioma is a common central nervous system cancer, ranking the 6^th^ cause in cancer-associated deaths worldwide [1, 2]. At present, the main treatment strategies for glioma contain surgery, radiotherapy and chemotherapy [3]. Even so, the prognosis of patients with high-grade glioma is still far from satisfactory [4]. Moreover, people have gradually realized that the occurrence and development of glioma are extremely complex processes [2]. Thus, it is urgent to uncover the molecular mechanism of glioma development and search for new molecular markers for the diagnosis and therapy of glioma [5].

Circular RNAs (circRNAs) are a family of single-stranded closed non-coding RNAs (ncRNAs) with neither 5’-terminal nor 3’-terminal poly A tails [6, 7]. CircRNAs can regulate gene abundance through functioning as the sponges for microRNAs (miRNAs) [8]. It has been manifested that circRNAs act as vital players in the carcinogenesis of multiple diseases, including glioma. For instance, circ_0007534 contributed to the malignancy of glioma by sponging miR-761 and elevating ZIC5 [9]. Circ_0012129 facilitated glioma cell growth and invasion by targeting miR-661 [10]. Circ_0079593 predicted poor prognosis and aggravated tumor cell malignant behaviors in glioma with the involvement of miR-182 and miR-433 [11]. As a member of circRNAs, circRFX3 (also termed as hsa_circ_0001836) has been verified to be upregulated in glioma patients [12]. Nevertheless, the precise functions of circRFX3 in glioma progression are entirely unknown.

MiRNAs, the small ncRNAs, are verified to alter gene expression through targeting the 3’ untranslated region (3’UTR) of target mRNAs [13]. Increasing literature has demonstrated that miRNAs play a regulatory role in glioma progression. For example, miR-936 acted as a tumor inhibitor in glioma by targeting CKS1 [14]. MiR-141-3p repressed glioma cell proliferation and facilitated apoptosis by modulation of ATF5 [15]. More importantly, miR-1179 acted as a glioblastoma inhibitor by interacting with E2F5 [16]. MiR-1229 restrained the development of glioma via modulating mTOR [17]. Whereas, the relationship between circRFX3 and miR-1179/miR-1229 is still unclear. Additionally, vasodilator stimulated phosphoprotein (VASP) participated in regulating glioma carcinogenesis by serving as the sponge of miR-605-3p [18]. Even so, there is no report on the interaction between miR-1179/miR-1229 and VASP in glioma.

In this paper, the expression profiles of circRFX3, miR-1179, miR-1229 and VASP in glioma tissues and cells were determined. Moreover, their relationships in regulating glioma cell malignant phenotypes were investigated.

## Materials and methods

### Tissues acquisition

Following the work was approved by the Ethics Committee of Huaihe Hospital, Henan University and written informed consents were provided by the participants, 50 glioma tissues were harvested from the patients with glioma and 50 normal tissues were obtained from the patients with cerebral trauma at Huaihe Hospital, Henan University. The samples were saved at − 80 °C until use. The clinical pathological features of 50 glioma patients were presented in Table 1.

**Table 1:** Correlation between circRFX3 expression and the clinical pathological features of 50 glioma patients

Characteristic	All cases (n = 50)	circRFX3 expression		P-value
		High(n = 25)	Low(n = 25)	
Age (years)				0.563
< 50	20	11	9	
≥ 50	30	14	16	
Sex				0.774
Male	29	14	15	
Female	21	11	10	
Tumor size, cm				0.395
> 4.5	23	13	10	
≤ 4.5	27	12	15	
MGMT				0.001*
Unmeth	25	7	18	
Meth	25	18	7	
WHO grade				0.024*
I–II	24	8	16	
III–IV	26	17	9	
IDH mutation status				0.145
IDH-mutated	20	13	7	
IDH-wildtype	30	12	18	

Chi square test was used. *P < 0.05;

### Cell culture and actinomycin D treatment

Four IDH-wild type (WT) glioma cell lines (A172, U251, SHG44 and LN229) were bought from Procell (Wuhan, China) and human normal astrocyte cell line (NHA) was bought from Mingzhoubio (Ningbo, China). All cells were cultivated at 37 °C in Dulbecco’s modified Eagle’s medium (DMEM; Procell) mixed with 10% fetal bovine serum (FBS; Procell) and 1% penicillin–streptomycin solution (Procell) in a humid incubator including 5% CO_2_.

To block transcription, A172 and U251 cells were exposed to Actinomycin D (2 μg/mL; Sigma-Aldrich, St. Louis, MO, USA) for 8 h, 16 h and 24 h. Afterward, the levels of circRFX3 and RFX3 mRNA were examined using the quantitative real-time polymerase chain reaction (qRT-PCR) analysis mentioned next.

### RNA extraction, RNase R treatment and qRT-PCR experiment

Total RNA was extracted with TRIzol reagent (Sigma-Aldrich) and quantified using NanoDrop 2000c spectrophotometer (Thermo Fisher Scientific, Waltham, MA, USA). RNase R treatment was performed on total RNA utilizing RNase R (3U/μg; Solarbio, Beijing, China) at 37 °C. Next, HiScript® II Reverse Transcriptase Kit (Vazyme, Nanjing, China) or miRNA 1st Strand cDNA Synthesis Kit (Vazyme) was adopted to reversely transcribe RNAs into cDNAs. Thereafter, qRT-PCR reaction was manipulated utilizing AceQ Universal SYBR qPCR Master Mix (Vazyme) and relevant primers (Sangon Biotech, Shanghai, China) on an ABI 7500 Real-Time PCR system (Applied Biosystems, Foster City, CA, USA). The results were computed with the 2^−ΔΔCt^ strategy. GAPDH and U6 served as controls. The primers were all designed by Sangon Biotech and the senquences were listed as follows: circRFX3: (F: 5’-TATGTAAATGATGGGGGTGGAGA-3’ and R: 5’-TTCCATAGCATTGACAACCATCT-3’); RFX3: (F: 5’-CACAGGCTCGACAGTGACC-3’ and R: 5’-GCACAGTCTGTACCTGCTGTA-3’); miR-1179: (F: 5’-GCGGAAGCATTCTTTCATT-3’ and R: 5’-CAAGGGCTCGACTCCTGTTC-3’); miR-1229: (F: 5’-CTCTCACCACTGCCCTC-3’ and R: 5’-CAAGGGCTCGACTCCTGTTC-3’); VASP: (F: 5’-TTCTTCGGTGACCACTTCCG-3’ and R: 5’-TCACCCTCTGTAGGTCCGAG-3’); GAPDH: (F: 5’-GAAGGTGAAGGTCGGAGTC-3’ and R: 5’-GAAGATGGTGATGGGATTTC-3’); U6: (F: 5’-CGCTTCGGCACATATACTA-3’ and R: 5’-CGCTTCACGAATTTGCGTGTCA-3’).

### Cell transfection

Short hairpin RNA targeting circRFX3 (sh-circRFX3#1, 5’-ATACTCATTTATCTTCTCCAC-3’, sh-circRFX3#2, 5’-ATTTATCTTCTCCACCCCCAT-3’ and sh-circRFX3#3, 5’-TCATTTATCTTCTCCACCCCC-3’) and scramble control (sh-NC), miR-1179 mimic (miR-1179), miR-1229 mimic (miR-1229) and mimic control (miR-NC), miR-1179 inhibitor (anti-miR-1179), miR-1229 inhibitor (anti-miR-1229) and inhibitor control (anti-NC), the overexpression vector of VASP (VASP) and control vector were synthesized by Sangon Biotech. These synthetic oligonucleotides or vectors were transfected into A172 and U251 cells through the usage of Lipofectamine 2000 (Invitrogen, Carlsbad, CA, USA).

### Cell Counting Kit-8 (CCK-8) assay

The viability of A172 and U251 cells was tested by CCK-8 kit (Sigma-Aldrich). In brief, the transfected cells (1 × 10^4^ cells/well) were seeded into the 96-well plates and cultured for 24 h, 48 h and 72 h. Then 10 μL CCK-8 solution was supplemented into each well and incubated for 4 h. The absorption at 450 nm was measured utilizing a microplate reader (Potenov, Beijing, China).

### Colony formation assay

The transfected A172 and U251 cells (300 cells/well) were plated into 6-well plates. After cultivation for 14 days, the colonies were washed with PBS (Sigma-Aldrich), fixed in 4% paraformaldehyde (Sigma-Aldrich) and then dyed with 0.1% crystal violet (Solarbio). The colonies with > 50 cells were imaged and quantified.

### Transwell assay

Transwell inserts (Corning Incorporated, Corning, NY, USA) precoated with or without Matrigel (Solarbio) were used for the determination of cell invasion and migration, respectively. Briefly, a total of 1 × 10^4^ A172 and U251 cells suspended in 200 μL serum-free medium were added into the upper compartments, while 600 μL medium containing 10% FBS (Procell) was added into the bottom compartments. After 16 h (for migration) or 18 h (for invasion) of incubation, the cells on the top of membranes were removed and the migrated/invaded cells were treated with 4% paraformaldehyde (Sigma-Aldrich) and stained by 0.1% crystal violet (Solarbio). An inverted microscope (Olympus, Tokyo, Japan) was used for capturing images with the magnification of 100 × . The average numbers of migrated or invaded cells were obtained by counting cells in five random areas.

### Flow cytometry analysis

The apoptosis capacity of A172 and U251 cells was tested with Annexin V-fluorescein isothiocyanate (FITC)/propidium iodide (PI) Apoptosis Detection Kit (Vazyme) in line with the manufacturers’ instructions. The results were evaluated with flow cytometry (Beckman Coulter, Atlanta, GA, USA).

### RNA pull-down assay

3’-biotinylated circRFX3 (Bio-circRFX3) or oligo probe (Bio-NC) was transfected into A172 and U251 cells and maintained for 24 h. After that, the cell lysates were interacted with streptavidin-coated magnetic beads (Invitrogen) for 2 h to generate probe-coated beads. At last, circRFX3-bound RNAs were isolated and subjected to the aforementioned qRT-PCR analysis for the abundance of miR-1179, miR-1229, miR-515-5p, miR-549 and miR-587.

### Dual-luciferase reporter assay

The sequences of circRFX3 or 3’UTR of VASP including the wild-type or mutant binding sites of miR-1179 and miR-1229 were amplified and cloned into pmirGLO plasmid (Promega, Fitchburg, WI, USA) to generate the luciferase plasmids. Then the constructed vectors (circRFX3-wt, circRFX3-mut, VASP-3’UTR-WT and VASP-3’UTR-MUT) were transfected into A172 and U251 cells together with miR-1179, miR-1229 or miR-NC using Lipofectamine 2000 (Invitrogen). At last, the luciferase activity was determined using the Dual-Luciferase Reporter Assay Kit (Promega).

### RNA immunoprecipitation (RIP) assay

RIP assay was done using Imprint® RNA Immunoprecipitation Kit (Sigma-Aldrich). In brief, after A172 and U251 cells were lysed in RIP buffer, cell extracts were collected and then cultivated with magnetic beads coated with anti-immunoglobulin G (anti-IgG) or anti-Argonaute-2 (anti-Ago2). Thereafter, the co-precipitated RNAs were isolated and subjected to qRT-PCR test for the enrichment of circRFX3, miR-1179 and miR-1229.

### Western blot assay

The extraction of total protein was performed using RIPA buffer (Solarbio). The BCA Protein Quantification Kit (Vazyme) was employed for protein quantification. Next, 20 μg proteins were split via sodium dodecyl sulfonate-polyacrylamide gel (Solarbio) and blotted onto 10% polyvinylidene difluoride membranes (Sigma-Aldrich). After that, the membranes were mixed in 5% slim milk for 1 h at indoor temperature and then incubated with primary antibodies against GAPDH (ab9485; Abcam, Cambridge, MA, USA) or VASP (bs229624; Abcam) overnight at 4 °C followed by interaction with secondary antibody (ab205719; Abcam) for 1.5 h at indoor temperature. The ECL kit (Vazyme) was employed for chemiluminescence visualizing.

### Murine xenograft model

The animal experiments were approved by the Ethics Committee of Animal Research of Huaihe Hospital, Henan University. The male nude mice were purchased from Beijing Vital River Laboratory Animal Technology Co., Ltd. (Beijing, China). A total of 1 × 10^6^ A172 cells were subcutaneously administrated into the flank of the mice. After that, tumor volume was examined once a week and computed with: volume (mm^3^) = length × width^2^ × 0.5. After 5 weeks, the mice were euthanized using 5% isoflurane for 3 min and the weight of neoplasms was examined. The neoplasms were stored at − 80 °C for further analyses.

### Statistical analysis

The experiments were conducted triple times. The data were estimated with GraphPad Prism 7 software and exhibited as mean ± standard deviation. The survival curve of patients was generated by Kaplan–Meier plot and analyzed by log-rank test. Difference analysis was conducted using Student’s *t*-test or one-way analysis of variance (ANOVA). It was considered as significant if *P* < 0.05.

## Results

### CircRFX3 level was elevated in glioma tissues and cell lines

**Fig. 1:**
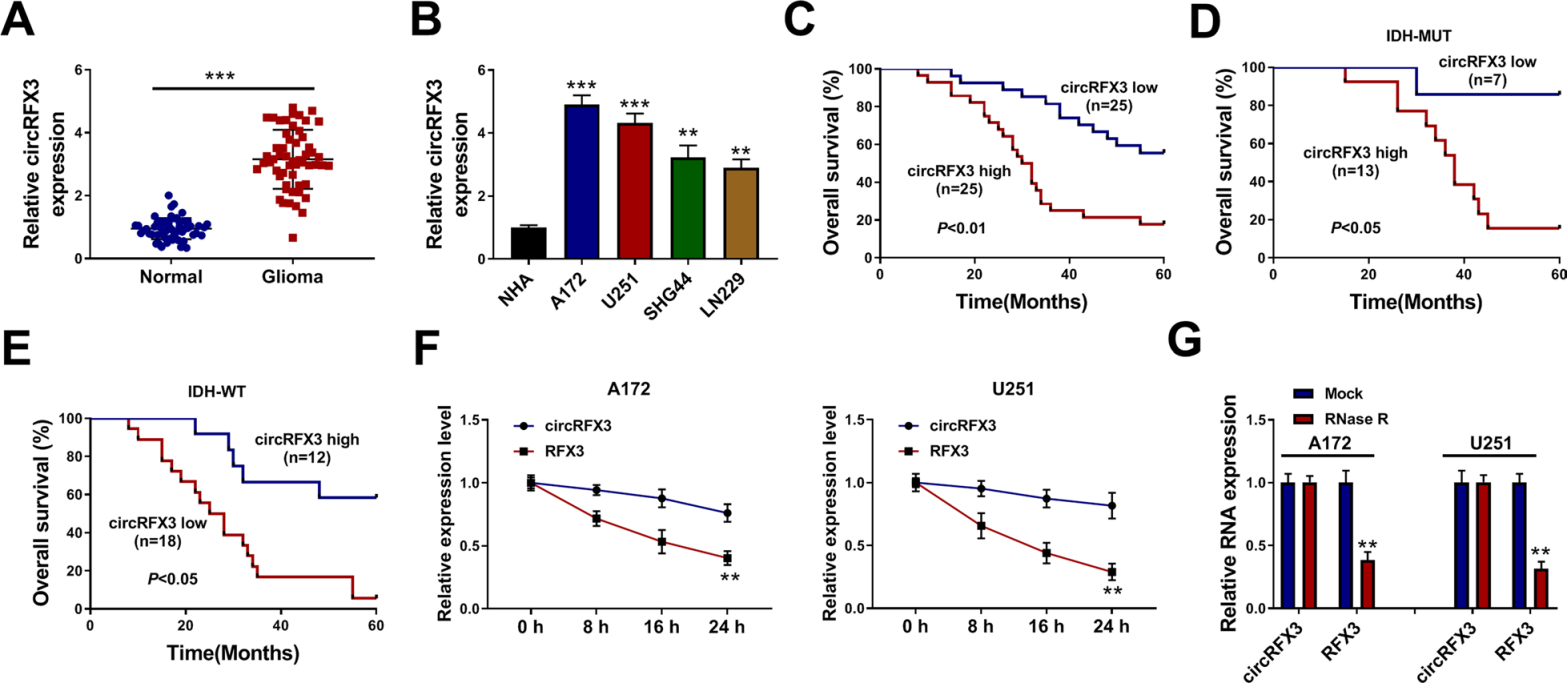
CircRFX3 was upregulated in glioma tissues and cells. **A** and **B** The expression level of circRFX3 in glioma tissues, glioma cells (A172, U251, SHG44 and LN229) and normal tissues and cells (NHA) was determined by qRT-PCR assay. **C** The overall survival of glioma patients was analyzed using Kaplan–Meier curve according to circRFX3 level. **D** and **E** The association between the overall survival of glioma patients in IDH-MUT/IDH-WT groups and circRFX3 level was analyzed. **F** After A172 and U251 cells were exposed to Actinomycin D for 0, 8, 16 and 24 h, the levels of circRFX3 and RFX3 in A172 and U251 cells were measured by qRT-PCR assay. **G** After total RNA in A172 and U251 cells was treated with or without RNase R, circRFX3 and RFX3 levels were determined by qRT-PCR assay. **P < 0.01, ***P < 0.001

To clarify the functions of circRFX3 in glioma progression, the expression level of circRFX3 in glioma tissues and normal tissues was firstly determined by qRT-PCR assay. The results showed that circRFX3 was highly expressed in glioma tissues compared to normal tissues (Fig. 1A). Then we determined the expression level of circRFX3 in glioma cell lines (A172, U251, SHG44 and LN229) and NHA cell line, showing that circRFX3 level was remarkably increased in glioma cells compared to NHA cells (Fig. 1B). A172 and U251 cell lines were selected for further experiments for the higher expression of circRFX3 in these two cell lines compared to SHG44 and LN229 cell lines (Fig. 1B). Additionally, according to the median of circRFX3 expression, the glioma patients were divided into two groups: high circRFX3 group (n = 25) and low circRFX3 group (n = 25). Our results showed that patients with high circRFX3 expression had a lower survival rate than patients with low circRFX3 expression (Fig. 1C). Moreover, our results showed that the patients with high circRFX3 exhibited worse overall survival rate even within the identical IDH status (both IDH-MUT and IDH-WT groups) (Fig. 1D and E). These results indicated that circRFX3 might be a prognostic marker for glioma. Thereafter, the characteristics of circRFX3 were determined by Actinomycin D assay and RNase R assay. As demonstrated by Actinomycin D assay, circRFX3 had a longer half-life than linear RFX3 (Fig. 1F). RNase R assay exhibited that circRFX3 was resistant to RNase R treatment, while RFX3 was markedly digested by RNase R (Fig. 1G). Collectively, circRFX3 was stable and might be involved in the progression of glioma.

### Knockdown of circRFX3 suppressed glioma cell viability, colony formation, migration and invasion and promoted apoptosis

**Fig. 2:**
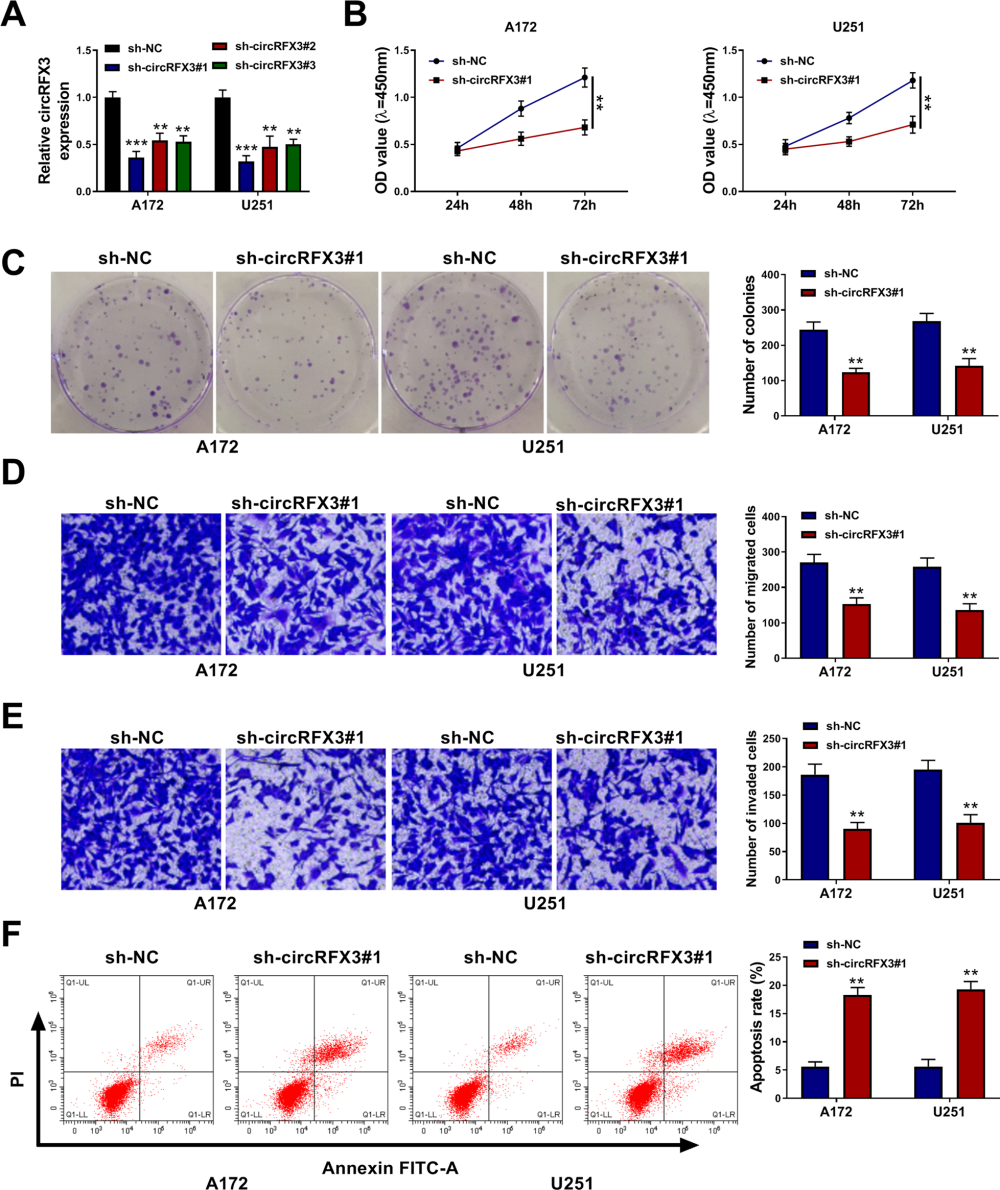
Effects of circRFX3 knockdown on glioma cell viability, colony formation, migration, invasion and apoptosis. **A** The expression level of circRFX3 in A172 and U251 cells transfected with sh-NC, sh-circRFX3#1, sh-circRFX3#2 or sh-circRFX3#3 was determined by qRT-PCR assay. (B-F) A172 and U251 cells were transfected with sh-circRFX3#1 or sh-NC. **B** The viability of A172 and U251 cells was assessed by CCK-8 assay. **C** The colony formation of A172 and U251 cells was evaluated by colony formation assay. **D** and **E** The migration and invasion of A172 and U251 cells were tested by transwell assay. **F** The apoptosis of A172 and U251 cells was analyzed by flow cytometry analysis. **P < 0.01, ***P < 0.001

To explore the exact roles of circRFX3 in glioma progression, loss-of-function experiments were conducted by transfecting sh-circRFX3#1, sh-circRFX3#2 or sh-circRFX3#3 into A172 and U251 cells. As shown in Fig. 2A, the transfection of sh-circRFX3#1, sh-circRFX3#2 or sh-circRFX3#3 evidently reduced the level of circRFX3 in A172 and U251 cells compared to sh-NC groups. In view of the lower expression of circRFX3 in sh-circRFX3#1 transfected cells than sh-circRFX3#2 or sh-circRFX3#3 transfected cells, sh-circRFX3#1 was used for further experiments. CCK-8 assay indicated that circRFX3 silencing conspicuously repressed the viability of A172 and U251 cells compared to sh-NC groups (Fig. 2B). The results of colony formation assay showed that circRFX3 knockdown led to a marked suppression in the colony formation capacity of A172 and U251 cells (Fig. 2C). As demonstrated by transwell assay, the migration and invasion of A172 and U251 cells were apparently inhibited by the deficiency of circRFX3 compared to control groups (Fig. 2D and E). Moreover, flow cytometry analysis exhibited that circRNF3 silencing obviously facilitated the apoptosis of A172 and U251 cells compared to sh-NC control groups (Fig. 2F). To summarize, circRFX3 knockdown restrained the malignant biological behaviors of glioma cells.

### CircRFX3 functioned as the sponge for miR-1179 and miR-1229

**Fig. 3:**
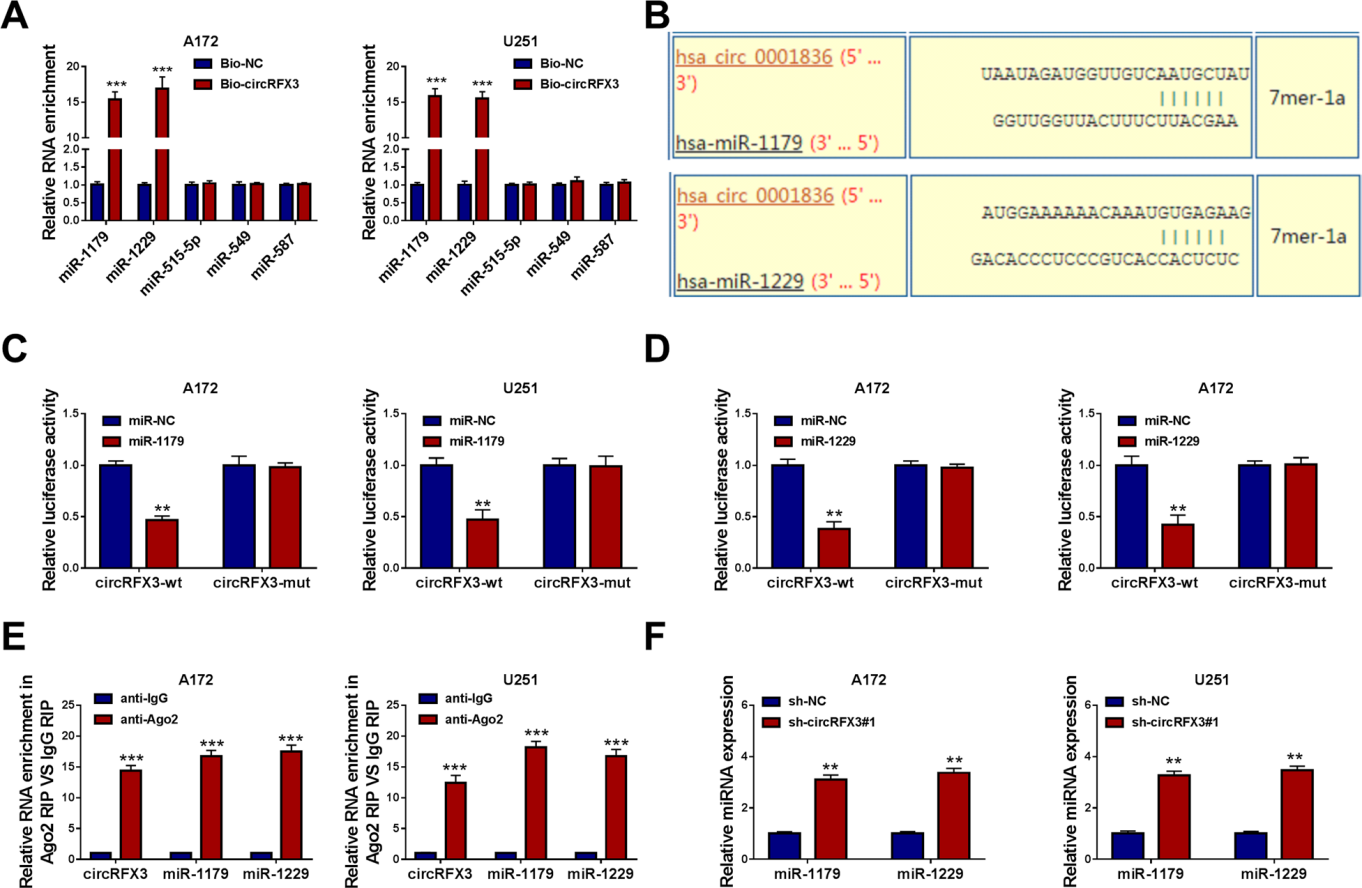
CircRFX3 directly targeted miR-1179 and miR-1229. **A** The levels of miR-1179, miR-1229, miR-515-5p, miR-549 and miR-587 in the streptavidin magnetic beads pulled down by Bio-circRFX3 or Bio-NC were determined by qRT-PCR assay. **B** The complementary sequences between circRFX3 (circ_0001836) and miR-1179/miR-1229 were shown. **C** and **D** The interaction between circRFX3 and miR-1179/miR-1229 was verified by dual-luciferase reporter assay. **E** The interaction between circRFX3 and miR-1179/miR-1229 was explored by RIP assay. **F** The expression levels of miR-1179 and miR-1229 in A172 and U251 cells transfected with sh-NC or sh-circRFX3#1 were determined by qRT-PCR assay. **P < 0.01, ***P < 0.001

Through analyzing online website circinteractome, we found there were 5 miRNAs (miR-1179, miR-1229, miR-515-5p, miR-549 and miR-587) contained the potential binding sites of circRFX3. Then we conducted RNA pull-down assay to analyze the interactions between circRFX3 and the candidate miRNAs. The results showed that Bio-circRFX3 could effectively pull down miR-1179 and miR-1229 in both A172 and U251 cells; thus, we selected miR-1179 and miR-1229 as our experimental objects (Fig. 3A). The predicted binding sites between circRFX3 (circ_0001836) and miR-1179/miR-1229 were presented in Fig. 3B. Next, dual-luciferase reporter assay and RIP assay were further conducted to verify the interaction between circRFX3 and miR-1179/miR-1229. The results of dual-luciferase reporter assay exhibited that the transfection of miR-1179 or miR-1229 markedly inhibited the luciferase activity of circRFX3-wt in A172 and U251 cells, while the luciferase activity of circRFX3-mut was not affected (Fig. 3C and D). RIP assay showed that circRFX3, miR-1179 and miR-1229 were drastically enriched in anti-Ago2 RIP group but not in anti-IgG group, further confirming the interaction between circRFX3 and miR-1179/miR-1229 (Fig. 3E). Furthermore, our results showed that sh-circRFX3#1 transfection remarkably increased the levels of miR-1179 and miR-1229 in A172 and U251 cells (Fig. 3F). All these results suggested circRFX3 negatively regulated miR-1179 and miR-1229 expression by direct interaction.

### MiR-1179 or miR-1229 inhibition reversed the effects of circRFX3 knockdown on glioma cell malignant behaviors

**Fig. 4:**
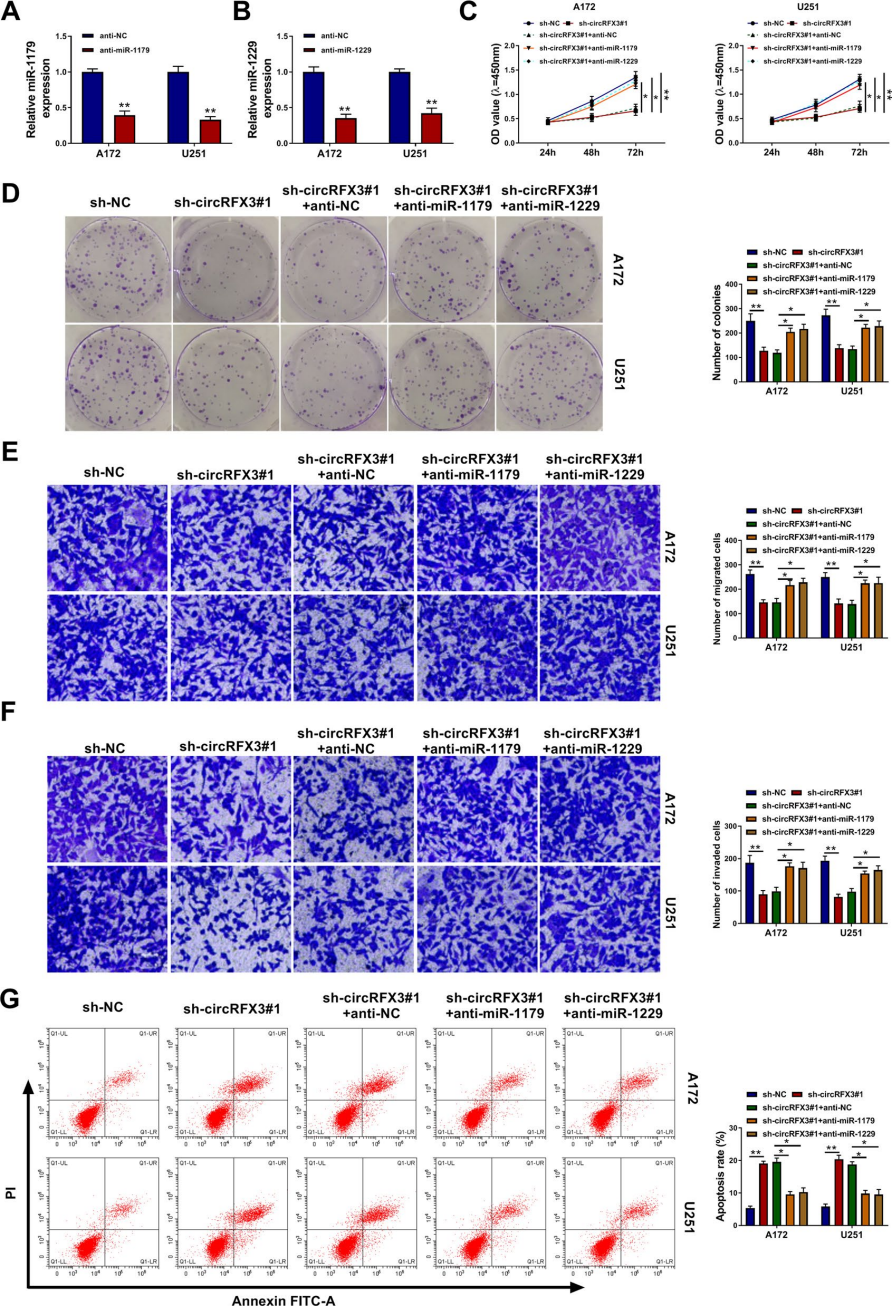
CircRFX3 silencing suppressed glioma cell progression by regulating miR-1179 and miR-1229. **A** The expression level of miR-1179 in A172 and U251 cells transfected with anti-miR-1179 or anti-NC was examined via qRT-PCR analysis. **B** The expression level of miR-1229 in A172 and U251 cells transfected with anti-miR-1229 or anti-NC was examined via qRT-PCR analysis. **C**–**G** After the transfection of sh-NC, sh-circRFX3#1, sh-circRFX3#1 + anti-NC, sh-circRFX3#1 + anti-miR-1179 or sh-circRFX3#1 + anti-miR-1229, the **C** viability, **D** colony formation, **E** and **F** migration and invasion, and **G** apoptosis of A172 and U251 cells were evaluated by CCK-8 assay, colony formation assay, transwell assay and flow cytometry analysis, respectively. *P < 0.05, **P < 0.01

As exhibited in Fig. 4A and B, anti-miR-1179 or anti-miR-1229 transfection evidently reduced miR-1179 level or miR-1229 level in both A172 and U251 cells compared to anti-NC groups. In order to elucidate the association between circRFX3 and miR-1179/miR-1229 in regulating glioma cell behaviors, A172 and U251 cells were transfected with sh-NC, sh-circRFX3#1, sh-circRFX3#1 + anti-NC, sh-circRFX3#1 + anti-miR-1179 or sh-circRFX3#1 + anti-miR-1229. The results of CCK-8 assay and colony formation assay presented that miR-1179 or miR-1229 inhibition strikingly reversed the impacts of circRFX3 silencing on the viability and colony formation of A172 and U251 cells (Fig. 4C and D). Transwell assay indicated that circRFX3 knockdown hampered cell migration and invasion in A172 and U251 cells, with miR-1179 or miR-1229 suppression abrogating the effects (Fig. 4E and F). In addition, the promotional effect of circRFX3 silencing on cell apoptosis in A172 and U251 cells was also ameliorated by decreasing miR-1179 or miR-1229 (Fig. 4G). These findings suggested that circRFX3 knockdown repressed the malignancy of glioma cells by targeting miR-1179 and miR-1229.

### VASP was the direct target gene of miR-1179 and miR-1229

**Fig. 5:**
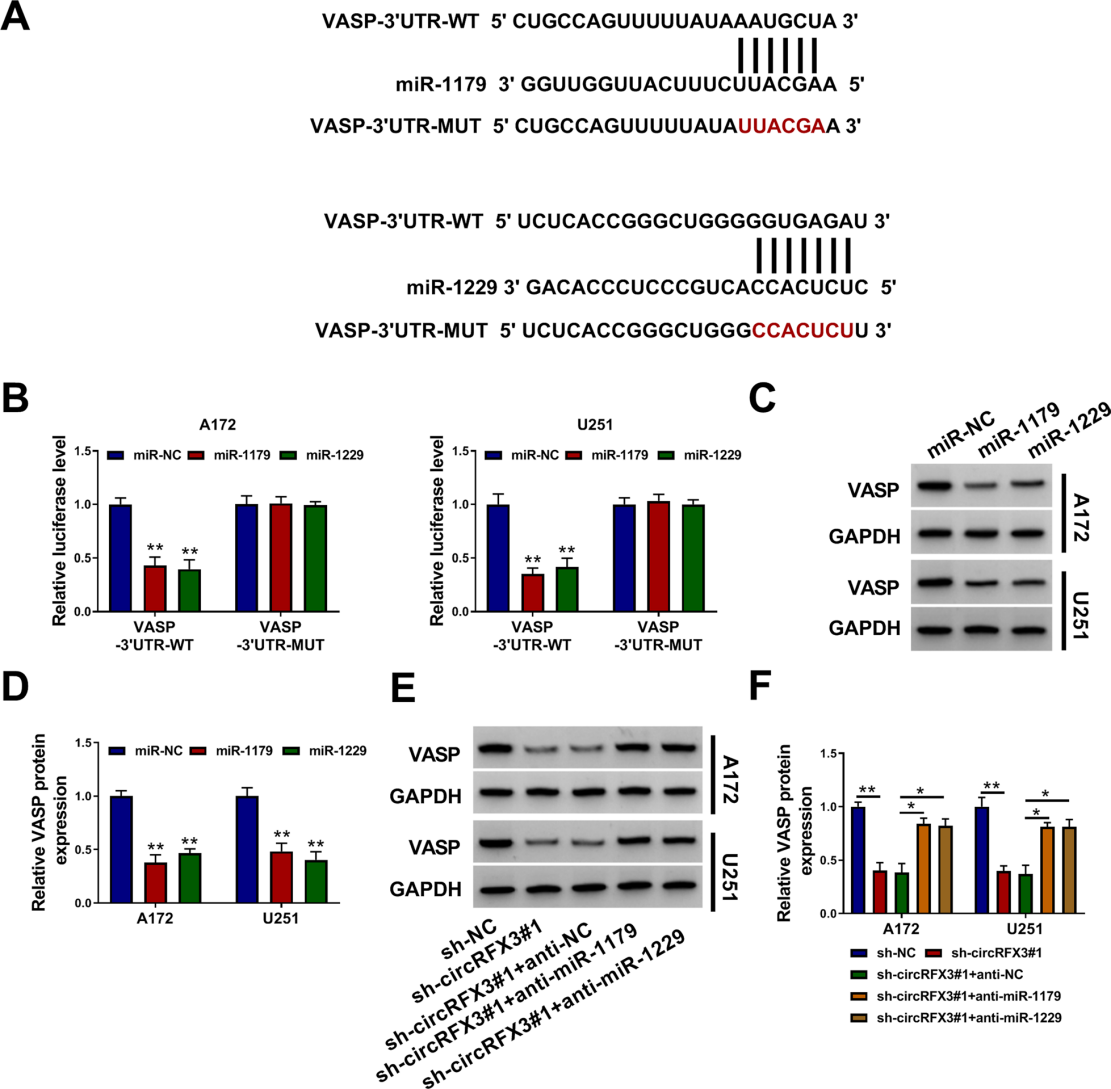
VASP acted as the target gene of miR-1179 and miR-1229. **A** The complementary sequences between VASP and miR-1179 or miR-1229 were shown. **B** The interaction between VASP and miR-1179/miR-1229 was verified using dual-luciferase reporter assay. **C** and **D** Following A172 and U251 cells were transfected with miR-NC, miR-1179 or miR-1229, the protein level of VASP was measured by western blot assay. **E** and **F** A172 and U251 cells were transfected with sh-NC, sh-circRFX3#1, sh-circRFX3#1 + anti-NC, sh-circRFX3#1 + anti-miR-1179 or sh-circRFX3#1 + anti-miR-1229, and then western blot assay was conducted for VASP protein level. *P < 0.05, **P < 0.01

By searching online software Targetscan, VASP was predicted to be the target gene of miR-1179 and miR-1229, and their binding sites were exhibited in Fig. 5A. Dual-luciferase reporter assay showed that miR-1179 or miR-1229 transfection apparently repressed the luciferase activity of VASP-3’UTR-WT in A172 and U251 cells, but there was no change in the luciferase activity of VASP-3’UTR-MUT, further demonstrating the interaction between VASP and miR-1179/miR-1229 (Fig. 5B). Moreover, we observed that miR-1179 or miR-1229 transfection led to a distinct reduction in VASP protein level in both A172 and U251 cells when compared to miR-NC control groups (Fig. 5C and D). Additionally, the results of western blot assay showed that circRFX3 silencing notably decreased the protein level of VASP in A172 and U251 cells, whereas miR-1179 or miR-1229 inhibition restored the effects (Fig. 5E and F). All these findings illustrated that circRFX3 knockdown decreased VASP expression by sponging miR-1179 and miR-1229.

### VASP overexpression reverted the effects of circRFX3 knockdown on glioma cell viability, colony formation, migration, invasion and apoptosis

**Fig. 6:**
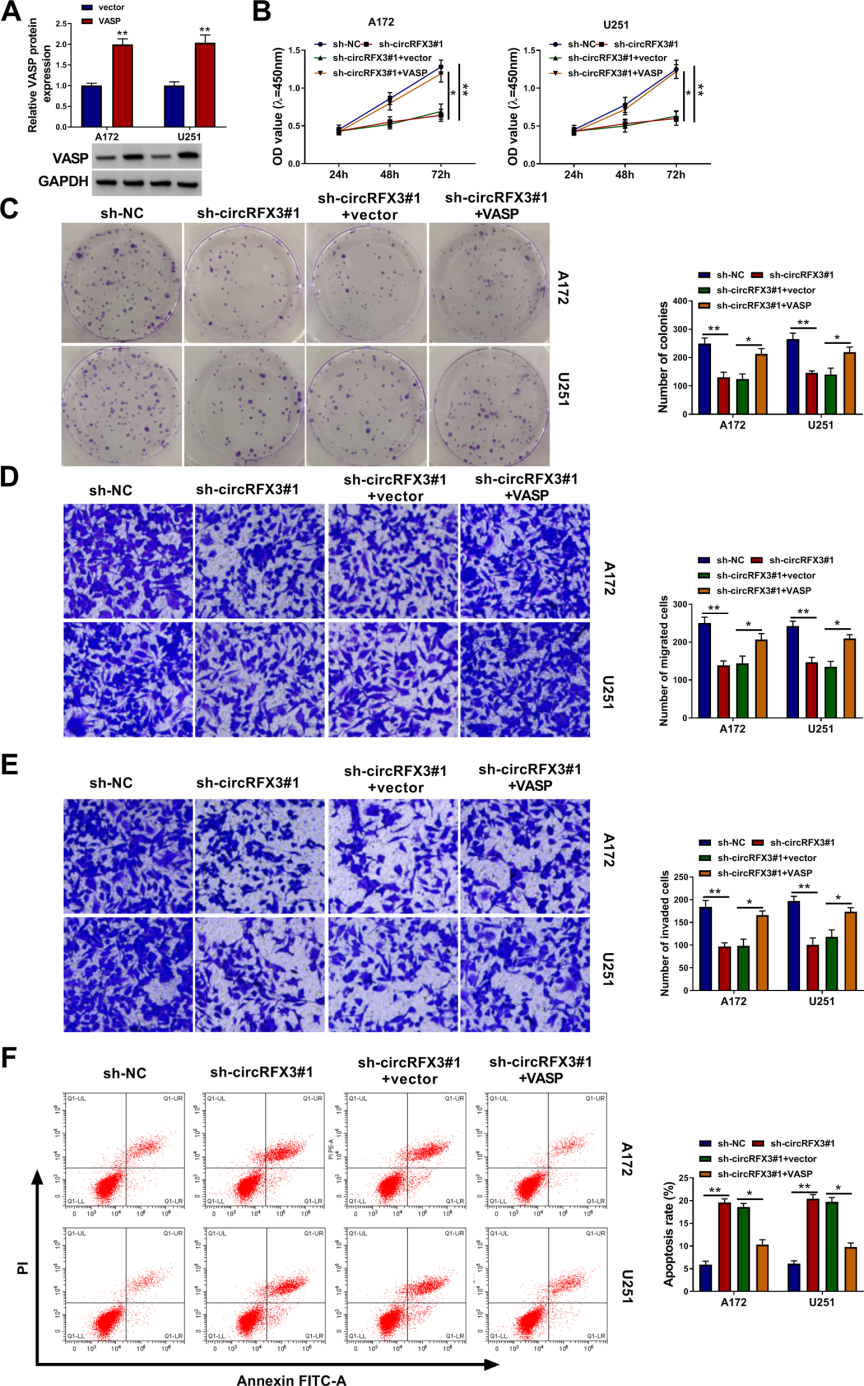
Overexpression of VASP abrogated the effects of circRFX3 silencing on glioma cell biological behaviors. **A** The protein level of VASP in vector or VASP transfected A172 and U251 cells was examined by western blot assay. **B**–**F** After the transfection of sh-NC, sh-circRFX3#1, sh-circRFX3#1 + vector or sh-circRFX3#1 + VASP, the **B** viability, **C** colony formation, **D** and **E** migration and invasion, and **F** apoptosis of A172 and U251 cells were evaluated by CCK-8 assay, colony formation assay, transwell assay and flow cytometry analysis, respectively. *P < 0.05, **P < 0.01

As presented in Fig. 6A, VASP was successfully transfected into A172 and U251 cells, as suggested by the upregulation of VASP protein level after VASP transfection. Next, A172 and U251 cells were administrated with sh-NC, sh-circRFX3#1, sh-circRFX3#1 + vector or sh-circRFX3#1 + VASP to explore the association between circRFX3 and VASP in glioma cell progression. As illustrated by CCK-8 assay, colony formation assay, transwell assay and flow cytometry analysis, the inhibitory effects on cell viability, colony formation, migration and invasion and the promotional effect on cell apoptosis in A172 and U251 cells mediated by circRFX3 knockdown were abolished by elevating VASP (Fig. 6B–F). All these data demonstrated that circRFX3 knockdown could repress the malignant phenotypes of glioma cells by regulating VASP.

### CircRFX3 knockdown restrained tumor growth of glioma in vivo

**Fig. 7:**
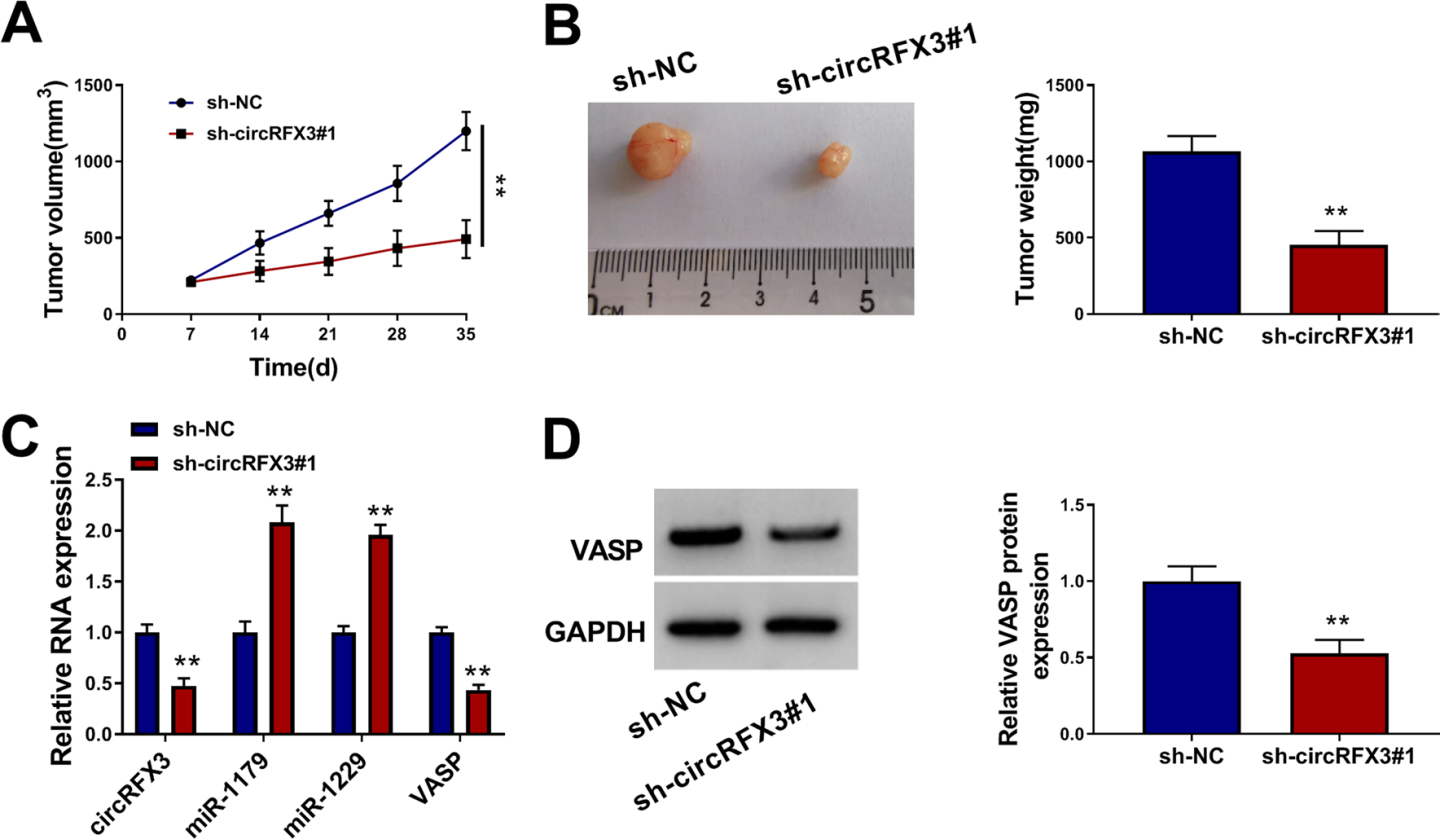
Silencing of circRFX3 blocked tumorigenesis of glioma in vivo. **A** After sh-circRFX3#1 or sh-NC transfected A172 cells were injected into the mice, tumor volume was monitored every 7 days. **B** Tumor weight was examined on day 35. **C** The levels of circRFX3, miR-1179, miR-1229 and VASP mRNA in the collected tumor tissues were measured by qRT-PCR assay. **D** The protein level of VASP in the collected tumor tissues was measured using western blot assay. **P < 0.01

To investigate the roles of circRFX3 in tumorigenesis in vivo, the murine xenograft model assay was conducted. As exhibited in Fig. 7A and B, the xenograft tumors in sh-circRFX3#1 group exhibited a marked reduction in tumor volume and tumor weight compared to sh-NC group. Moreover, we found that the levels of circRFX3, VASP mRNA and VASP protein were decreased and the levels of miR-1179 and miR-1229 were increased in the xenograft tumor tissues harvested from sh-circRFX3#1 group compared to control group (Fig. 7C and D). All these data indicated that circRFX3 could facilitate glioma progression in vivo.

## Discussion

Uncontrolled proliferation and migration of glioma cells are deemed as vital reasons for the poor prognosis of patients [19, 20]. The abnormal expression of circRNAs can function as tumor oncogenes or suppressors through regulating cell proliferation, migration and invasion [21]. Thus, we investigated the roles of circRFX3 in glioma. Our results verified that circRFX3 aggravated glioma cell growth and metastasis and alleviated apoptosis. Moreover, the feedback loop of circRFX3-miR-1179/miR-1229-VASP was discovered in glioma progression.

The functions of circRNAs in tumors have attached much attention due to the development of high-throughput sequencing technology and bioinformatics analysis [7]. Multiple circRNAs, such as circ-TTBK2 [22], circ-ABCB10 [23] and circ_0001730 [24], have demonstrated to play a crucial role in glioma via acting as competitive endogenous RNAs (ceRNAs). In the present research, the functional roles of circRFX3 were firstly investigated. CircRFX3 was highly expressed in glioma tissues and cells. Moreover, the high level of circRFX3 indicated the poor overall survival of glioma patients. Functionally, circRFX3 deficiency hampered glioma cell viability, colony formation and motility and accelerated apoptosis in vitro. In addition, murine xenograft model assay exhibited that circRFX3 silencing restrained the tumorigenicity of glioma in vivo. Taken together, circRFX3 exerted its role as a tumor promoter in glioma.

Subsequently, the underlying mechanisms of circRFX3 in glioma development were investigated. We confirmed that circRFX3 functioned as the sponges for miR-1179 and miR-1229 to positively regulate VASP expression. MiR-1179 has been claimed to be linked to the progression of human tumors, such as pancreatic cancer [25], breast cancer (BC) [26] and gastric cancer (GC) [27]. MiR-1229 also played crucial roles in the development of diverse tumors, such as BC [28], colorectal cancer [29] and GC [30]. Furthermore, miR-1179 and miR-1229 could regulate cell proliferation, cell cycle process and motility in glioma [16, 17]. Herein, we determined the association between circRFX3 and miR-1179/miR-1229. Our results showed that miR-1179 or miR-1229 inhibition ameliorated the suppressive impacts of circRFX3 knockdown on cell growth and metastasis and the promotional impact on apoptosis in glioma cells, illustrating that circRFX3 could modulate the malignant phenotypes of glioma cells by sponging miR-1179/miR-1229. Additionally, VASP elevation overturned the influences of circRFX3 silencing on glioma cell proliferation, motility and apoptosis, suggesting that circRFX3 stimulated glioma cell progression via elevating VASP expression.

## Conclusion

Altogether, circRFX3 level was raised in glioma and associated with poor prognosis. Functional and mechanical experiments indicated that circRFX3 promoted glioma cell growth and motility and repressed apoptosis by altering miR-1179/miR-1229-VASP axis. The results might offer a novel biomarker and therapeutic target for glioma.

## Data Availability

All data generated or analyzed during this study are included in this article.

## Abbreviations

circRNAs: Circular RNAs
VASP: Vasodilator stimulated phosphoprotein
